# Dual gut hormone receptor agonists for diabetes and obesity

**DOI:** 10.1172/JCI167952

**Published:** 2023-02-01

**Authors:** Joseph Bass, Matthias H. Tschöp, Lisa R. Beutler

**Affiliations:** 1 Northwestern University Feinberg School of Medicine, Department of Medicine, Division of Endocrinology, Metabolism and Molecular Medicine, Chicago, Illinois, USA.; 2 Helmholtz Munich, Neuherberg, Germany.; 3 Technische Universität München, Munich, Germany.

## Introduction

The epidemics of obesity and type 2 diabetes mellitus (T2DM) pose enormous threats to human health. Weight loss of at least 5%–10% attenuates the severity of these disorders and their complications. Diet and lifestyle modification are necessary but not sufficient for sustained weight loss in most people. Historically, antiobesity medications have been modestly effective, with numerous adverse side effects. Bariatric surgery offers sustained weight loss and potential remission of T2DM, but this treatment is impractical for large populations and may be associated with serious surgical and other complications, such as malabsorption and micronutrient deficiencies. The development of analogs of the gut peptide glucagon-like peptide 1 (GLP1) suggested considerable potential for gut hormones for treatment of T2DM and obesity. This culminated with regulatory approval of the GLP1 receptor (GLP1R) agonist semaglutide, which leads to clinically significant weight loss and improves T2DM control in most patients. The discovery of polyagonist drugs that activate multiple gut-brain pathways promises to further transform the management of obesity and T2DM. Recently, the gastric inhibitory polypeptide receptor/GLP1R (GIPR/GLP1R) co-agonist, tirzepatide, became the first polyagonist approved for the treatment of T2DM; it induces a staggering 22% average weight loss in patients with obesity (1). As the development of gut hormone dual- and tri-agonists progresses, there is hope for treatment of obesity and T2DM that rivals the efficacy of bariatric surgery.

## GLP1 and diabetes and obesity treatment

Obesity accounts for the vast majority of T2DM risk. While effective treatments for T2DM have existed for decades, some of these (e.g., insulin, sulfonylureas, thiazolidinediones) promote weight gain. The development of safe and effective antiobesity therapies has lagged significantly. Moreover, the FDA standard of 5% weight loss was barely attained by many approved drugs. This began to change when investigators first sought to harness the therapeutic potential of gut hormones, which mediate communication between peripheral organs and the brain. The incretin hormone GLP1 was discovered as a cleavage product of preproglucagon produced by L cells in the small intestine to enhance postprandial insulin secretion and reduce blood glucose (2–4). Native GLP1 is rapidly cleaved and inactivated by the protease dipeptidyl peptidase IV, which initially limited its clinical utility. The isolation of the protease-resistant GLP1R peptide agonist exendin-4 from Gila monster venom led to the development of exenatide, a synthetic form of exendin-4, which became the first GLP1R agonist approved for T2DM management in 2005 (5). Efforts since then have focused on prolonging GLP1 agonist activity through structural modifications (6). Liraglutide, a GLP1 analog approved in 2010 for T2DM, became the first gut hormone therapy approved for obesity treatment when administered at a higher dose (7). The long-acting GLP1R agonist semaglutide was approved for T2DM management in 2017 and for obesity management in 2021, with weight loss averaging 15% in clinical trials (8).

## Discovery of gut hormone co-agonists for obesity and diabetes

While GLP1 analogs were the first to make it to market, owing largely to the serendipitous discovery of exendin-4, GLP1 is one of many hormones that modulates glucose metabolism and regulates appetite. Although glucagon has largely been portrayed as the canonical counterregulatory glucose control hormone, which acts via the glucagon receptor to raise blood glucose, it also exerts lipolytic, thermogenic, and anorexigenic actions (9). To assess whether glucagon receptor activation could yield metabolic benefit if combined with simultaneous incretin receptor activity to mitigate its hyperglycemic effects, a groundbreaking endeavor was undertaken to develop novel antidiabetic and antiobesity medications termed polyagonists — single molecules that act on multiple receptors involved in metabolic homeostasis (10). A glucagon receptor/GLP1R dual agonist was the first polyagonist discovered in 2009 (11). However, the class of GIPR/GLP1R co-agonists was the first to realize the translational potential of polyagonism. GIP was initially isolated from intestinal extracts and shown to have a potent insulinotropic effect (12, 13). More recently, long-acting GIPR monoagonists were found to reduce body weight in obese mice (14). The first unimolecular GIPR/GLP1R co-agonist was reported in 2013 (15). This peptide was rationally engineered based on the sequences of the individual peptides and has balanced activity at the two receptors. It dose-dependently improved glycemia and obesity in rodents and primates beyond GLP1 monoagonism. Dual GIPR/GLP1R co-agonists were shown to be safe and efficacious in humans (16), and recently tirzepatide was the first GIPR/GLP1R co-agonist to receive regulatory approval for the treatment T2DM in 2022. Clinical trials have demonstrated that tirzepatide leads to an average of 22.5% weight loss in obese, nondiabetic patients (1).

Today, numerous other gut hormone polyagonists are in the drug development pipeline for obesity and T2DM. Glucagon receptor/GLP1R co-agonists were the first gut peptide polyagonists to be developed by engineering glucagon to render it a less specific ligand for the glucagon receptor, while also targeting GLP1R (11). In rodents, these compounds dramatically reduced body weight and improved glycemia. Long-term data from people treated with glucagon-containing dual agonists is emerging. A phase II trial of the glucagon receptor/GLP1R dual agonist cotadutide resulted in 5% weight loss at 54 weeks, a far cry from the dramatic weight loss seen with tirzepatide or semaglutide (17). However, the activity of cotadutide at the glucagon receptor, which is strongly expressed on hepatocytes, may improve nonalcoholic fatty liver disease (NAFLD) beyond its effects on body weight. If confirmed in additional trials, this would be of great clinical significance, given the rising prevalence of NAFLD and nonalcoholic steatohepatitis as global drivers of cirrhosis and liver transplant. Finally, high-potency triagonists, which simultaneously target the human GIPR, GLP1R, and glucagon receptors in a balanced manner, have been found in rodents and primates to have a greater effect on metabolic parameters than even dual GIPR/GLP1R co-agonists (18). Clinical trials evaluating efficacy and safety of dual and triagonists in T2DM, obesity, and NAFLD are underway and have been reviewed recently (19).

## Dissecting molecular mechanisms of gut hormone co-agonists

**Figure 1:**
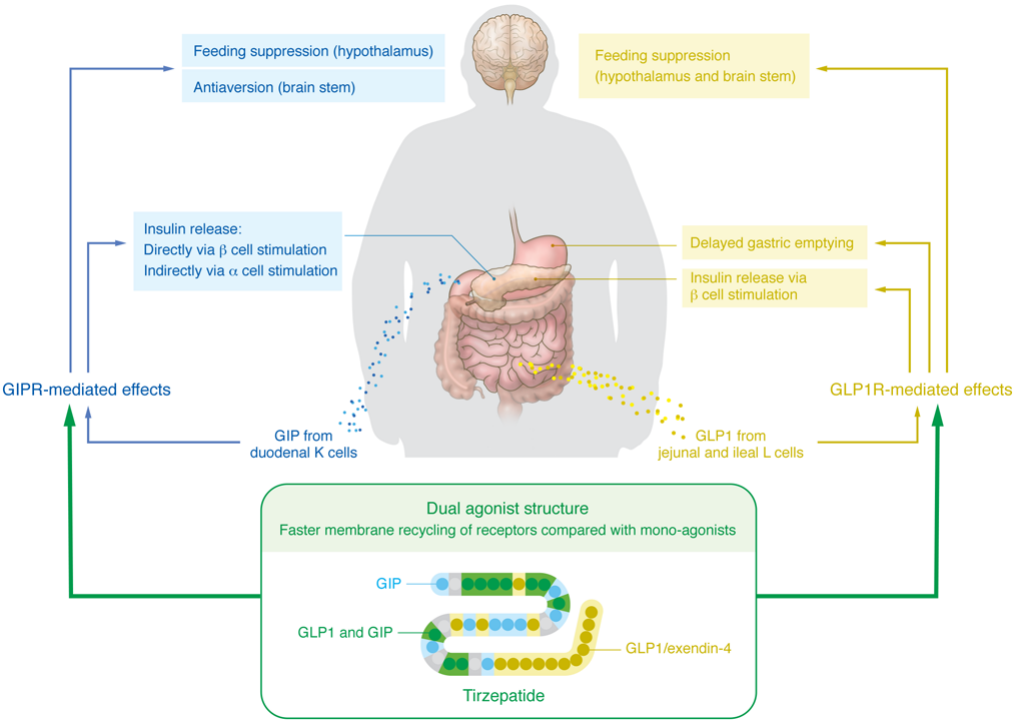
Established and proposed mechanisms underlying the glucoregulatory and weight loss effects of GIP/GLP1R co-agonism.

The discovery of and regulatory approval process for gut hormone polyagonists have outpaced our understanding of their underlying mechanisms of action. How do receptors expressed in peripheral organs and the brain mediate the complex metabolic and behavioral effects of these drugs? Sophisticated mouse genetic, molecular, and neuroscience approaches have begun to unravel how these molecules may exert synergistic effects on metabolism via their activity at different receptors. Determining the exact mechanisms by which GIPR agonism potentiates the effects of GLP1R agonism is a particularly active area investigation, given the remarkable clinical success of tirzepatide (Figure 1). GIP potentiates glucose-stimulated insulin release, primarily via binding to GIPR on pancreatic β cells through a mechanism involving K-ATP channels — a process distinct from GLP1 potentiation of insulin release (20). GIPR on α cells also indirectly promotes insulin release via stimulation of glucagon secretion (21).

As noted above, long-acting GIP analogs induce dose-dependent weight loss, an effect that is abrogated in GIPR-knockout mice (14). Thus, a component of enhanced GIPR/GLP1R dual agonist efficacy relative to GLP1 monoagonism may be related to independent effects at the individual receptors. The exact location of the GIPR required for GIP agonist–induced weight loss is uncertain, though recent evidence shows that CNS GIPRs are essential for this effect. Specifically, GIPR in hypothalamic feeding centers appear to be involved (22). Interestingly, a dose of GIPR agonist that has no effect on body weight enhances GLP1 analog–induced weight loss (15), suggesting that the mechanisms underlying incretin polyagonist efficacy may be more complex than a purely additive effect of the two hormones on their receptors. At least two intriguing hypotheses to explain this synergy have been proposed. First, through biased agonism at the GLP1R, tirzepatide induces faster recycling of internalized GLP1R compared with GLP1R monoagonism (23). Second, evidence suggests a global antiaversive effect of GIPR agonism, which could allow for enhanced GLP1R agonism while minimizing nausea and other gastrointestinal side effects that limit GLP1R agonist tolerability (24, 25). The GIPRs studied in antiaversion studies are located in the brain stem, separate from the hypothalamic receptors that may mediate the direct effect of GIPR agonism on food intake. Finally, GIPR antagonism and global GIPR knockout in mice has also been reported to protect against obesity, and GIPR antagonists are also in the antiobesity drug development pipeline (26). Ultimately, elucidating the in vivo effects of GIPR agonism versus antagonism may offer insight into how each affects T2DM, obesity, and other metabolic diseases.

The role of glucagon agonism in the treatment of metabolic disease. The mechanisms underlying the anorectic effects of glucagon are poorly understood, but they are abrogated by vagotomy and may involve hepatic futile cycling and/or secretion of FGF21 (9). Others have shown that the beneficial effects of the glucagon receptor/GLP1R co-agonist cotadutide on blood glucose and bodyweight are predominantly mediated by the GLP1R, while the effects on NAFLD require glucagon receptor activation (27). This finding may depend upon the potency of specific polyagonists at individual receptors. Given that glucagon receptor agonism stimulates hepatic gluconeogenesis, the long-term benefits and adverse consequences of polyagonists must be carefully analyzed.

## Conclusions and future work

The dual incretin agonist tirzepatide is the first pharmacological agent that rivals the efficacy of bariatric surgery. However, several important issues must be addressed. To date, nearly all GLP1 agonists and tirzepatide are administered as daily or weekly subcutaneous injections, with prolonged dose-escalation paradigms to minimize gastrointestinal side effects. This limits their use in some patients. Although an oral formulation of semaglutide coupled to an absorption enhancer is available, its bioavailability is low and it lacks the full glucose and body weight lowering effects compared with injected semaglutide. Therefore, the development of potent, small-molecule, orally bioavailable incretin receptor (poly-)agonists and allosteric modulators is an active area of drug development (19, 28). As the repertoire of gut-derived hormone receptor polyagonists expands, it will also be critical to address the issue of weight regain upon cessation of treatment (29). Moreover, common gastrointestinal side effects and potential risks for gall stones, pancreatitis, and thyroid cancer must continue to be evaluated. The development of tirzepatide supports the concept of combination therapy for T2DM and obesity, similar to combination therapies for hypertension and other chronic diseases. The rapid expansion of molecular tools to unravel the basic mechanisms underlying gut-brain axis control of feeding and metabolism, coupled with efforts to harness these pathways with the next generation of engineered polyagonists, will further advance the development of precision medicine for obesity, T2DM, and related diseases.

## Version 1 02/01/2023

Electronic publication
